# Detection of Dengue Virus in *Aedes aegypti* Mosquitoes, Dubai, United Arab Emirates 

**DOI:** 10.3201/eid3208.260324

**Published:** 2026-08

**Authors:** Norbert Nowotny, Noushad Karuvantevida, Elmahi Eltilib Gubran, Naseem Mohammed Rafee, Adel Abdulla AlKarrani, Ghanim Al Hammadi, Nasna Nassir, Dia Advani, Mohammed Uddin, Jeremy V. Camp, Hanan Alsuwaidi, Alawi Alsheikh-Ali

**Affiliations:** University of Veterinary Medicine Vienna, Vienna, Austria (N. Nowotny); Mohammed Bin Rashid University of Medicine and Health Sciences, Dubai Health, Dubai, United Arab Emirates (N. Nowotny, N. Karuvantevida, N. Nassir, D. Advani, M. Uddin, H. Alsuwaidi, A. Alsheikh-Ali); Dubai Municipality, Dubai (E.E. Gubran, N.M. Rafee, A.A. AlKarrani, G. Al Hammadi); GenomeArc Inc., Mississauga, Ontario, Canada (M. Uddin); Medical University of Vienna, Vienna (J.V. Camp); Dubai Health Authority, Dubai (A. Alsheikh-Ali).

**Keywords:** dengue virus, viruses, *Aedes aegypti*, *Anopheles stephensi*, entomological surveillance, mosquitoes, vector-borne infections, mosquito-borne, Dengue, Chikungunya, Zika, Yellow fever, Rift valley fever, Alkhurma hemorrhagic fever, West Nile Virus, *Plasmodium*, Malaria, Dubai, United Arab Emirates, Arabian Peninsula

## Abstract

We performed a 1-year mosquito survey in Dubai, United Arab Emirates, after a dengue virus outbreak in 2024. We collected 1,598 *Aedes aegypti* mosquitoes and detected dengue virus in 5 pools of <20 mosquitoes. Our findings underscore the importance of sustained vector surveillance for dengue virus in arid urban environments.

During the past few decades, mosquitoborne diseases have increased worldwide, partly because of the spread of competent mosquito vectors. Changes in climate are among the primary factors contributing to the expanded ranges of arbovirus vector mosquitoes into temperate zones (*1*). Meanwhile, arid regions with extremely hot summers, such as the Gulf countries of the Arabian Peninsula, were not colonized by invasive mosquitoes until recently, likely because of increased urbanization (*2*).

Dengue fever is a mosquitoborne disease caused by dengue virus (DENV) and monitored through regional and international surveillance systems. Increased dengue activity, including autochthonous cases, was reported in the United Arab Emirates (UAE) after periods of increased rainfall in April 2024 (*3*). To monitor local mosquito species and identify circulating mosquitoborne viruses, we conducted a mosquito survey in Dubai, UAE. 

## The Study

To investigate the spread and distribution pattern of *Aedes* *aegypti* mosquitoes in the city of Dubai, we initiated a targeted mosquito survey. We evaluated the trapped mosquitoes for the presence of mosquitoborne human pathogens by using molecular techniques.

Mosquito trapping was part of an enhanced Dubai Municipality Public Health Services Department mosquito surveillance program. We started the entomologic survey on June 20, 2024, and continued it for 1 year. Our trapping method was opportunistic, designed to sample *Ae. aegypti* mosquitoes in urban areas, specifically targeting labor camps, construction sites, residential communities, and green spaces including parks, stables, ponds and lakes, and gardens. 

We placed BG-Sentinel mosquito traps (Biogents, https://us-shop.biogents.com) without a light source throughout the city of Dubai combined with CO_2_ lures. We conducted trapping over 3 days at 60 sites, each sampled 1–33 times (mean 4.3 times, median 2 times), with 1–7 sites (mean 1.9 sites, median 1 site) sampled per trapping period. We collected and transferred the trap contents to the Public Health Pest Control Section laboratory of Dubai Municipality. We identified *Ae. aegypti* and *Anopheles stephensi* mosquitoes morphologically (*4*; World Health Organization, https://iris.who.int/handle/10665/334210) and identified other mosquitoes to genus level. We pooled <20 mosquitoes per vial and froze the vials at –50°C. 

We homogenized mosquito pools in 500 µL of phosphate buffered saline with stainless steel beads (Benchmark Scientific, https://www.benchmarkscientific.com) on a Bead Mill homogenizer (OMNI International, https://www.fishersci.com). We extracted viral nucleic acid from mosquitoes by using a Quick-RNA Viral Kit (Zymo Research, https://www.zymoresearch.com) according to manufacturer instructions. We screened *Ae. aegypti* mosquitoes for dengue, chikungunya, Zika, yellow fever, Rift Valley fever, and Alkhurma hemorrhagic fever virus nucleic acids by using virus-specific quantitative reverse transcription PCR (qRT-PCR) kits (altona Diagnostics, https://altona-diagnostics.com). We retested and serotyped all samples positive for DENV by using the RealStar Dengue Type qRT-PCR Kit 1.0 (altona Diagnostics). We screened *Culex* spp., *Aedes*, and *Ochlerotatus* spp. mosquitoes for West Nile, Rift valley fever, and Alkhurma hemorragic fever virus nucleic acids by using the corresponding RealStar qRT-PCR Kits (altona Diagnostics). We also screened *An. stephensi* mosquitoes for *Plasmodia* parasites by using the RealStar Malaria Screen & Type PCR kit 1.0 (altona Diagnostics). 

**Figure 1:**
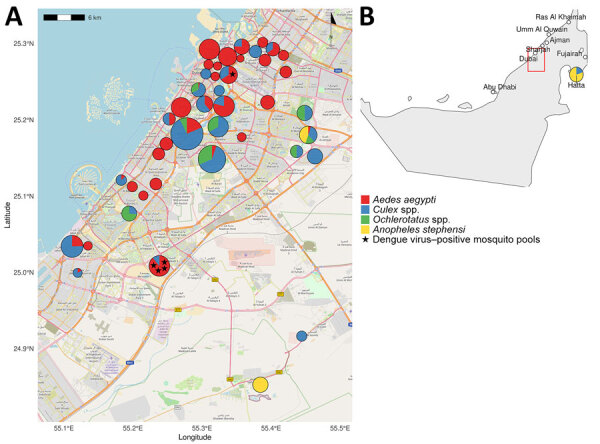
Geographic distribution of trapped mosquitoes in study of dengue virus in *Aedes aegypti* mosquitoes, Dubai, United Arab Emirates. A) Of the 60 sampling sites, all except 3 were located within the city of Dubai; 2 were slightly outside the city borders and 1 in Hatta, a mountainous exclave of the emirate of Dubai ≈135 km east of the city of Dubai. Sampling sites were combined for better presentation if within 1 km of each other. B) Capital cities of the 7 emirates and the study sites indicated. Mosquito genera and species are shown by different colors; the size of the circle represents the relative number of trapped mosquitoes.

**Figure 2:**
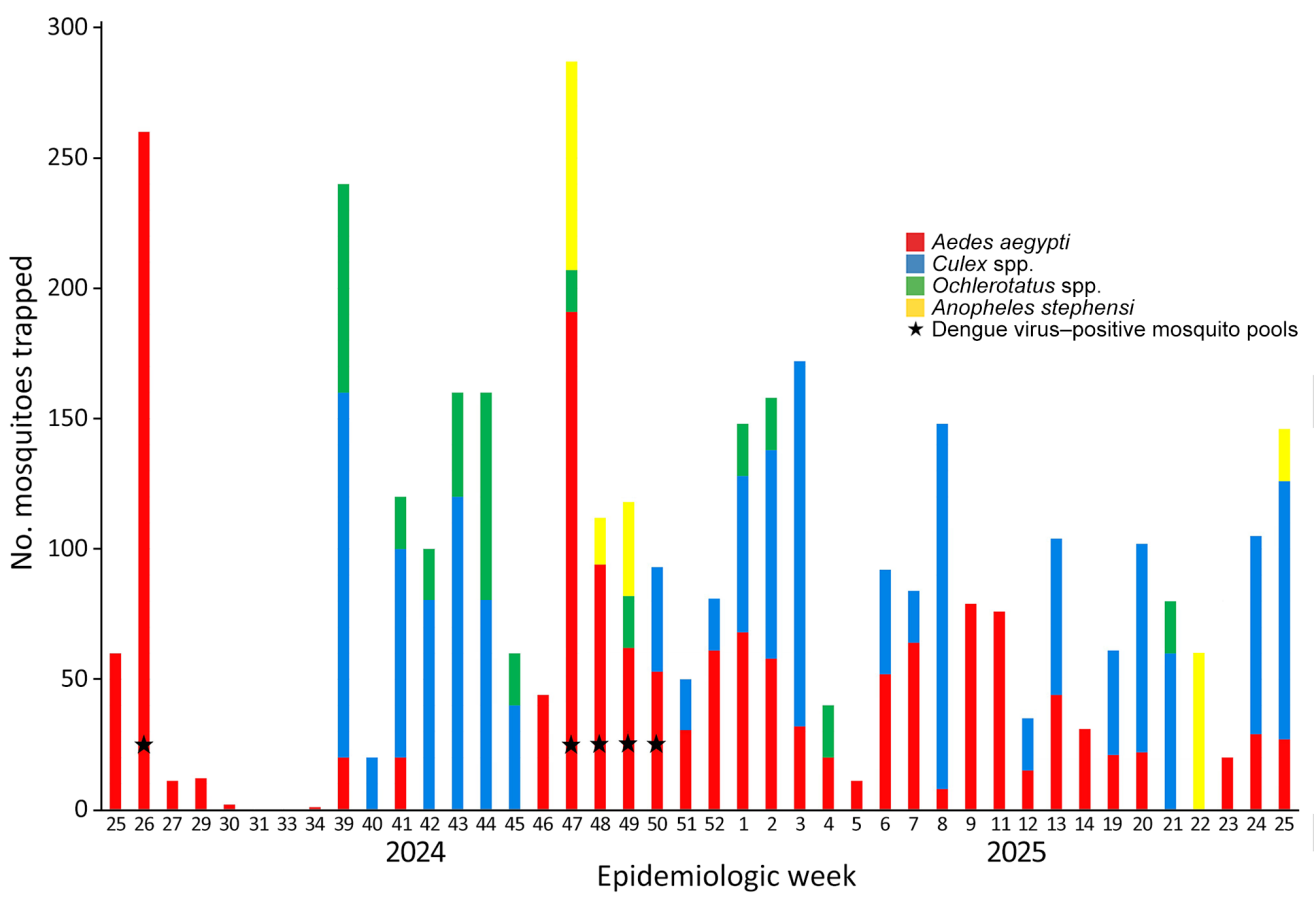
Number of mosquitos trapped in study of dengue virus in *Aedes aegypti* mosquitoes, Dubai, United Arab Emirates, June 2024–June 2025. We used BG Sentinel traps (Biogents, https://us-shop.biogents.com) with CO_2_ at various sites around Dubai to capture mosquitoes. Mosquitoes were trapped over 3-day trap-periods, with 1–7 (mean 1.9, median 1) sites per period. *Aedes (Stegomyia) aegypti* and *Anopheles stephensi* mosquitoes were typed to species level. In epidemiologic weeks 35–38 of 2024 and 15–18 of 2025, no mosquitoes were trapped.

We used a bias-corrected maximum likelihood estimate of minimum infection rate on the basis of variable size pooling with the package binGroup v2.2-1 in R version 4.3.3 (The R Project for Statistical Computing, https://www.r-project.org). We collected a total of 3,743 adult mosquitoes over 263 trapping periods: 1,598 *Ae. aegypti*, 1,555 *Culex* spp., 376 *Ochlerotatus* spp., and 214 *An. stephensi*. We found *Ae. aegypti* mosquitoes throughout Dubai, mainly collected from labor camps, construction sites, and green spaces, whereas we detected *An. stephensi* mosquitoes in limited numbers in periurban areas with aquatic habitats (Figure 1). We observed seasonal variability in the number of mosquitoes collected, with the lowest number collected during the summer months (Figure 2).

We detected DENV in 5 of 151 *Ae. aegypti* pools: 3 pools with DENV-1 and 2 with DENV-2. The field minimum infection rate was 3.1 (95% CI 0.4–5.9)/1,000 mosquitoes . We detected DENV-2 in the last week of June (epidemiologic week 26 of 2024) near the main Dubai airport (Figure 2). We detected both DENV-1 and DENV-2 from mid-November through mid-December (epidemiologic weeks 47–50 of 2024) in the southwest Al Hebiah 5 district (Figure 2). We did not detect any other viruses in the other mosquito pools, and we did not find *Plasmodia* parasites in any of the mosquito pools with *An. stephensi* mosquitoes.

In most of the Gulf countries, the workforce is recruited from countries in which dengue, chikungunya, Zika, yellow fever, Rift Valley fever, or West Nile virus are endemic, and imported cases were previously reported in the Arabian Peninsula. Dengue fever outbreaks in the Arabian Peninsula have occurred in Yemen and the Jeddah and Mecca region of Saudi Arabia (*5*). Oman, which borders UAE, experienced its first autochthonous dengue outbreak during December 2018 through mid-March 2019, with 59 reported cases (*6*). A second autochthonous outbreak occurred from mid-March through mid-April 2022 and involved 169 locally acquired cases (*7*). 

In the UAE, neither *Aedes* (*Stegomyia*) mosquitoes nor locally acquired dengue cases were reported in recent decades (*5*). Historically, there was evidence of *Ae. aegypti* mosquitoes in UAE as early as 1944 (*8*). However, it is assumed that the populations of *Ae. aegypti* mosquitoes from older records would not have survived the liberal use of insecticides in the second half of the 1900s (*9*). We did not find *Ae. aegypti* mosquitoes in our entomologic survey conducted during January–May 2018 in the UAE (*10*). The first recent record of an *Ae. aegypti* mosquito in Dubai was in May 2016, identified by the Public Health Pest Control Section laboratory, Dubai Municipality, and confirmed by the British Natural History Museum. Other Gulf countries such as Qatar also reported recent, yet sporadic, evidence of *Ae. aegypti* mosquitoes (*11*). Regional and global species distribution models have indicated that urban areas in Gulf countries are suitable for *Ae. aegypti* mosquitoes (*12*). Likely, the increased DENV activity we observed was driven by a recently established *Ae. aegypti* population in Dubai.

In recent years, increased frequency of heavy rainfall with flooding and stagnant surface water were recorded worldwide including the Gulf countries, contributing to an increase in mosquito abundance and dengue cases (*13*). In addition, manmade water-containing vessels can serve as reservoirs for immature *Ae. aegypti* mosquitoes and correlate with cases of dengue fever (*14*). Those sources of water in arid environments likely contribute to extended mosquito survival during dry periods (*15*). 

## Conclusions

Our results demonstrate the widespread distribution of *Ae. aegypti* mosquitoes in Dubai. We detected DENV-1 and DENV-2 in *Ae. aegypti* mosquitoes from 2 sampling locations within the urban area of Dubai, which coincided with a DENV-2 outbreak in humans (Dubai Health, unpub. data). Our findings demonstrate the value of integrated entomologic and molecular surveillance for early detection of mosquitoborne pathogens in arid urban settings. Future studies should establish seasonality and habitat associations to assist with mosquito control.

The presence of *Ae. aegypti* mosquitos in urban areas in the Gulf region necessitates steps to reduce or eliminate DENV transmission. Efforts should include educating the public on avoiding mosquitoes, surveillance and targeted mosquito control, and isolating infected individuals quickly to prevent spread. 
